# How understanding and application of drug-related legal instruments affects harm reduction interventions in Cambodia: a qualitative study

**DOI:** 10.1186/s12954-017-0167-9

**Published:** 2017-06-19

**Authors:** Sovannary Tuot, Chanrith Ngin, Khuondyla Pal, Sochenda Sou, Ghazal Sawez, Phylicia Morgan, Mony Srey, Tola Chan, Pheak Chhoun, Olga Golichenko, Sok Chamreun Choub, Siyan Yi

**Affiliations:** 1 KHANA Center for Population Health Research, Phnom Penh, Cambodia; 2 Asia Actions for Harm Reduction, KHANA, Phnom Penh, Cambodia; 3 Public Health Program, Touro University California, Vallejo, CA 94592 USA; 4 Royal University of Phnom Penh, Phnom Penh, Cambodia; 5 International HIV/AIDS Alliance, Brighton, UK; 6 KHANA, Phnom Penh, Cambodia

**Keywords:** Harm reduction, Law and policy application, Drug law and policy, Qualitative study, Cambodia

## Abstract

**Background:**

Harm reduction interventions in Cambodia face numerous obstacles because of conflicting understanding and interests and inconsistencies in the implementation by law enforcement officials. This study aims to examine how understanding and application of Drug Control Law (DCL) and Village/Commune Safety Policy (VCSP) affects harm reduction interventions in Cambodia from the standpoints of law enforcement officials, people who inject drugs and people who use drugs (PWID/PWUD), as well as other key stakeholders.

**Methods:**

This qualitative study was conducted in the capital city of Phnom Penh in 2015. We held five focus group discussions (FGDs) with groups of PWID/PWUD, police officers, Sangkat/commune officers, and local non-governmental organization (NGO) field staff. We also conducted ten key informant interviews (KIIs) with representatives from government agencies, donor agencies, and NGOs. FGDs and KIIs with Cambodian participants were transcribed in Khmer and translated into English. KIIs with foreign participants were transcribed in English. Transcripts were read and re-read to identify emerging themes, which were reviewed and refined to develop common and divergent patterns.

**Results:**

There was a huge gap between what the DCL and VCSP say and how law enforcement officers and PWID/PWUD understood them. The gap was also evident in how law enforcement officers implemented the DCL and VCSP. Harm reduction services, including health- and non-health-related interventions, were limited and challenged by unsupportive attitudes, misinterpretation of the DCL and VCSP, and the lack of full engagement with NGOs in the development of these instruments. The needs of PWID/PWUD in accessing health care services were not met due to misconduct of authorities while practicing the DCL and VCSP. Further, the misconduct and enforcement of the law and policy lead to increased social discrimination and physical abuses against PWID/PWUD.

**Conclusions:**

There is a lack of common understanding of the drug-related law and policy and their implications to harm reduction services among both law enforcement officers and PWID/PWUD. Thus, there is a need to mainstream and simplify the law and policy for better comprehension among these actors. To improve the quality and coverage of harm reduction interventions, the gap of understanding and enforcement of laws and policies should be narrowed, and coordination between the government and NGOs and other key stakeholders should be strengthened.

## Background

Globally, drug-related problems are increasing and becoming more intertwined with development issues [1]. To effectively combat drug problems, it requires “development-sensitive” drug control policies [1]. Research evidence presented in the World Drug Report 2016 indicates that “efforts to achieve the Sustainable Development Goals and to effectively address the world drug problems are complementary and mutually reinforcing” [1]. In other words, to address problems related to the use of illicit drugs, policies need to aim at “the overall social, economic and environmental development of communities” [1]. Many experts agree that punitive policies do not work to reduce health and socio-economic problems associated with drugs in the long run [2–4]. In Asia, for instance, evidence indicates that punitive policies and practices exacerbate drug use and consequent ills, and human rights-based approaches work better [5]. Suppressive policies and social discrimination could worsen the HIV epidemic among people who inject drugs (PWID) and people who use drugs (PWUD) since they are discouraged from carrying clean needles and syringes due to the fear of being arrested and may not seek health services due to stigmatization [4, 6]. Conversely, human rights-based and voluntary community-based approaches prove to be effective in preventing and treating drug use and related diseases [4, 6]. For example, in Malaysia, transforming compulsory detention centers into voluntary cure and care centers attracts more PWID/PWUD to receive health services [7].

In Cambodia, two major legal instruments have been enacted to combat drug trafficking and drug use, namely the Drug Control Law (DCL) [8] and the Village/Commune Safety Policy (VCSP) [9, 10]. The DCL, originally ratified in 1996 and periodically modified in 2005 and 2007, was lastly amended in 2012. This law essentially stipulates administrative and legal actions and punishment against illegal drug production, trafficking, and use. However, it embraces some harm reduction elements, including voluntary treatment, choice of treatment method, and sufficient treatment periods for PWID/PWUD. For instance, Article 105 allows the police to refer PWID/PWUD to a treatment program as an alternative to criminal prosecution.

Nonetheless, the DCL contains some ambiguities, inconsistencies, and controversies surrounding treatment of PWID/PWUD. While the police are entitled to send PWID/PWUD to a treatment program, the DCL does not provide clear guidance for prosecutors as to what treatment program and when to refer them to. Article 107 states that “forced treatment shall not be imposed unless there is a serious situation;” but it does not define the term “seriousness” or the level of “seriousness.” Articles 45 and 53 implicitly criminalize repeated use of drugs by PWID/PWUD by stating that “drug users who have already received forced treatment and rehabilitation and are caught using drugs again will face imprisonment from one to six months.” Further, Article 40 articulates that “a person in possession of drugs faces imprisonment from two to five years,” without specifying the amount of drugs carried. As a result, police officials have challenges in distinguishing between PWID/PWUD and drug dealers [11].

The VCSP, launched in 2010, aims to combat crimes such as robbery, drug trafficking, and other illegal activities such as prostitution and illegal gambling [9, 10]. This policy requests authorities to “cut off and eliminate production, dealing, and use of illegal drugs in villages and communes” as part of efforts to ensure “public security and order” [9]. However, it was classified as “red” legislation by human rights groups in 2012, meaning it violates the core principles of human rights [12]. Since the launch of the VCSP, law enforcement officers have extensively used the DCL as a legal basis to implement the VCSP with regards to crimes and violence in relation to drug trafficking and drug use [10]. Some articles of the VCSP (and the DCL) allow law enforcement officers to check PWID/PWUD for condoms, needles, and syringes and arrest them or force them to relocate. Violation of human rights and physical abuses of PWID/PWUD take place regularly [10], making PWID/PWUD live in fear of being arrested and/or removed from communities [10]. In many cases, PWID/PWUD were isolated from health, education, legal support, and other harm reduction services [10, 11]. Another repercussion of the VCSP was that besides the police’s suppressing approach, communities could report illegal activities, such as drug use, to local authorities [11, 13]. Also, parents could report and turn in their children who used illicit drugs to the police for rehabilitation.

When talking about and implementing the DCL and VCSP, law enforcement agencies, particularly local authorities in Cambodia, emphasize physical safety issues. This is reflected by the fact that treatment programs for PWID/PWUD are mostly conducted within compulsory rehabilitation centers, which are hardly different from prisons [14]. Also, this is reflected by a lack of community-based treatment approaches adopted by the government [14]. Moreover, the concepts of harm reduction and its implementation have not been well understood and accepted by the general public and law enforcement officers. The general public, and law enforcement officers, sometimes refer to harm reduction as needle and syringe program (NSP), while harm reduction embraces more aspects of health and human rights-related issues among PWID/PWUD [11]. In addition, in Cambodia, due to the perception that the risk of the transmission of HIV through injecting drug use is not as high as through unsafe sex, many law enforcement officers are not convinced that NSP is an effective means to prevent HIV transmission among PWID in Cambodia [11]. Their disbelief is exacerbated by a lack of evidence of the effectiveness of NSP in the Cambodian context [11].

The discrepancies in the understanding and application of drug-related legal instruments and harm reduction programs warrant further investigation into perspectives of concerned actors about these issues. Previous research mainly explored these issues from law enforcement perspectives, insufficiently addressing perspectives of PWID/PWUD and harm reduction practitioners [10, 11, 14, 15]. This study aimed to examine how the understanding and application of the DCL and VCSP affects harm reduction interventions in Cambodia from the standpoints of PWID/PWUD, harm reduction practitioners, law enforcement officials, and other key stakeholders.

In Cambodia, illicit drug use is on the rise [1]. The estimated numbers of PWID/PWUD in the country vary, ranging from 20,000 in 2012 [16] to 46,000 in the same year [10]. However, government estimates put the numbers of PWID/PWUD at 10,000 in 2012 and 16,600 in 2015, respectively [17, 18]. Despite the different estimates, there is a common agreement that drug-related health and socio-economic problems are increasing [1, 18]. In 2015, the police escalated law enforcement on 3061 drug-related cases (up from 1339 cases in 2014), arresting 7008 suspects (up from 3138 suspects in 2014) and seizing nearly 2000 kg of drugs [18]. Cambodia has a much less serious opiate/heroin and injecting problem than other Southeast Asian countries, and amphetamine-type stimulants (ATS) are a major drug problem [1]. This contextual issue particularly affects “harm reduction,” which here (and elsewhere) is mostly focused on needles and methadone [5, 11].

There have been various efforts to address drug-related issues in Cambodia, including rehabilitation programs [18] and community-based treatment and reintegration programs [19]. The government recognized and accepted harm reduction programs in 2003, when the HIV epidemic declined from its peak from 2% in 1998 to 1.2% in 2003 [20]. At that time, the epidemic shifted from the general population to the key populations (KPs), which include female sex workers (FSW), men who have sex with men (MSM), transgender women (TG), and PWID/PWUD [20]. PWID, whose total population in the country was estimated at around 1300, had one of the highest HIV prevalence estimates (24.8% in 2012) [16]. Eventually, the government recognized drug use as social and health problems and acknowledged the need to take a harm reduction approach in response to HIV among PWID in 2003, and officially launched the NSP in 2005. However, this transitional solution was mainly from the perspective of public health and was driven by the HIV response and funding; no new laws and policies were approved to formally support NSP operations. It was only in 2013 that the National Authority for Combating Drugs (NACD) started to draft policy and guiding principle for the operation of NSP [21], which was finally completed in 2014. The approaches to addressing drug related issues in Cambodia, both in the law and practices, are punitive.

Non-governmental stakeholders have active engagement in harm-reduction-related activities [11, 13]. The engagement includes providing technical and financial support to harm reduction programs as well as service delivery and policy advocacy. The main features of harm reduction implementation for PWID in Cambodia include NSP, HIV education, HIV counseling and testing, healthcare referrals, and free methadone. However, as we will show, the NSP program is not widely accepted because of, among other barriers, conflicts with the VCSP and DCL, the norm and belief in the communities, and the law enforcement, and therefore not being widely used since its approval in 2005. Moreover, the NSP program is unable to reach all PWID in communities and reportedly is not well understood and welcomed by some police officers and community members [11].

Rehabilitation centers are the most common service that law enforcement officers can use to refer arrested PWID/PWUD to [22, 23]. There are several centers, which are run by NGOs, the private sector and the government. Based on the DCL, there should be no forced treatment unless necessary. However, in practice, the referrals of PWID/PWUD to the centers are usually based on an agreement with parents or guardians of the PWID/PWUD or sometimes without an agreement of the PWID/PWUD or their parents or guardians. Physical abuses of PWID and PWUD were reported occurring in many centers.

Community-based treatment program, a more comprehensive approach being strengthened and scaled-up by the Ministry of Health (MoH), is an alternative to compulsory rehabilitation centers [19, 24, 25]. This program has been integrated into the general public healthcare system under the MoH with the support from the United Nations Office on Drug and Crimes (UNODC) and World Health Organization (WHO). The community-based treatment program in Cambodia uses multi-stakeholder collaboration to refer PWID/PWUD to community-based drug dependence treatment services [19, 24, 25]. The program includes comprehensive health and psychosocial care services provided by health centers and referral hospitals as well as social reintegration and support services provided by NGOs. Other distinctive features of the program are effective collaboration from law enforcement officers who are sensitized and trained to support the program and peer educators who conduct outreach, home visits, and counseling. Notably, in the community-based treatment program, methadone maintenance therapy (MMT) is not a priority since the bulk of clients mainly consume ATS.

After piloting in a province in 2012, the community-based treatment program was planned to be expanded nationwide in 2016 thanks to its effectiveness in treatment, reintegration, and skills training of clients [19, 22, 23, 26]. The MoH planned to integrate NSP in the community-based treatment at health centers and referral hospitals [11]. An assessment of the community-based intervention program in three provinces in 2015 pinpointed that its success relied on strong leadership and national commitment, meaningful involvement of clients, community participation, NGO engagement, and multi-sectoral collaboration and coordination between public health, public security, and NGO sectors [26]. Notwithstanding, the community-based treatment program encountered a number of shortcomings, including non-functioning of some services, compromised quality of care, and limited access, due to decreasing financial and technical aid and overloaded staff. Further, provision of care through public health facilities made clients reluctant to access services due to fear of arrest, stigmatization, or discrimination. Also, the program depended on NGOs to provide social support services, including vocational training, family support, and social reintegration. Thus, to make the community-based treatment program more effective and sustainable, these challenges need to be addressed.

Given the unique context of drug use and the specific approaches to harm reduction in Cambodia, it is crucial to examine how the understanding and application of the DCL and VCSP among the various stakeholders affect these approaches. This paper is intended to provide this insight.

## Methods

This qualitative study was carried out in the capital city of Phnom Penh in 2015. This city has more than 80% of the total PWID/PWUD in the country [16]. We chose focus group discussions (FGDs) and key informant interviews (KIIs) as qualitative tools to capture the understanding, implementation, and perceptions of the drug-related laws and policies by the stakeholders. These qualitative tools allowed the participants to express, perceive, and discuss their views. The number of FGDs was determined based on the key categories of the stakeholders. The number of KIIs was determined based on the relevance and diversity of the stakeholders. Attempts were made to capture the most relevant and diverse stakeholders.

We held five FGDs with PWID/PWUD, police officers, Sangkat/commune officials, and local NGO field staff. We also conducted KIIs with four government representatives each from NACD, National AIDS Authority (NAA), MoH, and Ministry of Interior (MoI)—two donor representatives from the United Nations Joint HIV/AIDS Programmes (UNAIDS) and WHO and four local NGO representatives (program-level staff who are working directly with PWID/PWUD).

The FGDs with PWID/PWUD were conducted at KHANA’s office, while the FGDs with police officers, Sangkat/commune officials, and local NGO field staff were held at their respective offices. The KIIs with other stakeholders were done at their respective offices. With permission from the participants, the FGDs and KIIs were recorded.

A stratified purposive sampling method was employed to recruit the study participants. First, Phnom Penh, which has the bulk of PWID/PWUD, was selected. Second, from Phnom Penh, three Khans/districts with a concentrated population of PWID and PWUD were selected. Finally, a Sangkat/commune with the most PWID/PWUD in each Khan/district was chosen. Nine PWID (two females), nine PWUD (two females), seven police officers, four Sangkat/commune officials, and nine local NGO field staff, who were residing or working in the selected Sangkats/communes, were chosen to participate in their respective FGDs. The participating PWID and PWUD were beneficiaries of and chosen by the participating NGOs. The criteria for recruiting PWID/PWUD included an age of 18 years old or above, with an attempt for gender balance. The participating police officers, Sangkat/commune officials, and NGO field staff were assigned by their superiors. The participating representatives from government institutions, donor agencies, and NGOs were selected based on their job responsibilities for drug-related issues.

The instruments used for FGDs and KIIs were developed based on the following topics: (1) knowledge, attitude, and engagement of participants in drug use, law, and policy development and implementation; harm reduction practices; (2) needs and challenges of PWID/PWUD to support and improve their living; (3) impacts of the law and policy and their implementation on PWID/PWUD; and (4) recommendations to improve harm reduction practices from all stakeholders. These topics were constructed based on the literature on harm reduction in Cambodia in particular [10–13] and in developing countries in general [5, 6, 14]. Draft instruments were consulted with a number of our partner NGOs working on harm reduction before being finalized.

The FGDs and KIIs with Cambodian participants were conducted and transcribed in Khmer and translated into English. The KIIs with foreign participants were held and transcribed in English. Transcripts were read and re-read to identify emerging themes, which were reviewed and refined to develop common and divergent patterns across the study participants. Data were coded and analyzed in accordance with the following themes: availability of harm reduction programs, awareness about and attitudes toward harm reduction and its implementation, engagement in development of and awareness about the DCL and VCSP, needs and challenges of PWID/PWUD, and factors enabling mistreatment of PWID/PWUD. The themes were related to all types of drugs that PWID/PWUD used, not specific to different drugs.

This study was approved by the National Ethics Committee for Health Research (NECHR Ref. 0371), Ministry of Health, Cambodia. Participation in this study was voluntary. We ensured the confidentiality of all information the participants provided by excluding their identity, other identifiable information, and personal information from the study documentation. The participants were verbally explained the study objectives and their participation rights. They had the rights to object, refuse to answer, or withdraw from the study at any time. They were ensured that their participation or non-participation would not render any consequence on them. All participants provided a verbal informed consent prior to the start of the FGDs or KIIs.

## Results

### Awareness about and attitudes toward harm reduction

Although policy makers and harm reduction service providers have recognized the criticality of harm reduction, its concept is not publicly well understood.

The awareness of harm reduction was limited and different among the participants—PWID/PWUD, governmental agencies, and non-governmental agencies. Grassroots implementation officers (police officers) reported that NSP motivated more drug use.

*“This NSP program motivates the continuation of drug use and may encourage new PWID/PWUD. The community usually complains about the danger that could be caused by the wasted needles and syringes around their houses.”- A police officer in FGD*

NGO participants pointed out the different levels of understanding and implementation of harm reduction among stakeholders. They also emphasized a need for sensitization training on related laws and policies for police officers.

*“For harm reduction in Cambodia, my understanding is that the policies and the implementation are not at the same level of understanding. The implementation is so challenging that we need to sensitize the police and train them to understand well any relevant laws and policies.”– An NGO representative in KII*

Moreover, there is an urgent need to have more and transparent funding allocation.

*“We had very limited resources invested into the harm reduction programs so far. Harm reduction funding allocation was not transparent. And partners (NGOs and donors) always complained about PWID and PWUD who always moved from one location to another.”– A government representative in KII*

### Engagement in development of and awareness about DCL and VCSP

Our results indicate that there was almost no evidence that the process of developing the DCL and VCSP was conducted in a consultative and participatory manner in which key stakeholders (especially beneficiaries and non-governmental agencies) were actively involved. None of the study participants, except one from the NACD who had been involved in the amendment, reported that they or their institutional representatives were granted rights or given an opportunity to engage in the process of developing these legal instruments.

*“During the process of developing the DCL, our organization proposed 12 points to amend this law, but our proposal was not seriously considered.”- An NGO representative in KII*

PWID/PWUD reported that they did not know of any applicable law or policy that the police used to deal with them or drug-related issues; although, the DCL and VCSP have long been adopted and executed. As a result of not knowing the legal instruments and associated rights, PWID and PWUD viewed themselves as unlawful citizens or criminals. Therefore, they rarely dared to complain about mistreatment, including physical abuses, by the authorities.

*“…Yes, the police are right to beat us because we use illicit drugs.”- A PWID in FGD*

Unlike the PWID/PWUD group, the majority of participants from the government agencies and NGOs reported that they were aware of the DCL and VCSP. However, their understanding of this law and policy was limited or vague. The ambiguity in the understanding of the law was evident among the participants, including law implementation officers (police) and non-governmental agencies, when they showed their disagreement on criminalization of PWID/PWUD. They had contrasting understanding about whether police officers and other levels of law implementation officers had to arrest PWID/PWUD.

*“We know that the VCSP was introduced in 2010, and also the drug law which sets criminalization against PWID/PWUD in at least one of its articles. We talked a lot with the MoI and the NACD about these policy and law as they hamper the implementation of the harm-reduction-related programs because of their strong statement of criminalization in their articles. Through this long talk and conversation, with the show of reality that harm reduction (and of course drug use) is not against the policy and law but rather contribute to achieve its objectives, we now arrive at a process to amend the articles [the article numbers were not mentioned] by redefining the drug use which should not be criminal.” – An NGO representative in KII*

*“I believe that using drugs is illegal and should be punished, at least one month in detention… For the VCSP, it means ‘no drug and crime’, which makes the policy sound a bit harsh for drug users. But, the policy should be harsh because the implementation of the VCSP is based on the DCL.”- A government representative in KII*

*“We feel that the police at the lowest level (post/commune police) are harsher than the higher level police (Khan/district police).”- PWID in FGD*

The findings show that many relevant stakeholders did not clearly understand the DCL, in particular when asked about such important articles as 40, 45, 53, 104, 105, and 107. The lack of understanding and clarity of interpretation led to the gaps in their implementation to a great extent.

*“…I don’t know what Article 40 is about, but when I see them (PWID/PWUD) carrying drugs with whatever amount, they are considered a criminal or drug dealer and can be arrested…If he/she is a PWID/PWUD, not a dealer, he/she should not have (unused) drugs with him/her…”- A police officer in FGD*

The limited awareness and understanding of the DCL and VCSP were reasonable since very limited formal training on or dissemination of the law and policy had been conducted, not even for implementing officers. Only one police officer in the study claimed to have attended a quarterly meeting at district level in which he was informed about the VCSP. Other local authorities learned about the DCL and VCSP from their monthly open and public meetings between themselves and local communities where they discussed the public service provision and community security, etc.

*“The lack of awareness of laws is at a great extent. We understand that some laws were created with good mechanisms for implementation, but basically it is based on the government’s political will and available resources. Generally, there is a huge gap between the law/policy and the enforcement because of interests involved in the laws. For example, some police officers practice according to the order of their big boss, which is sometimes not conforming to the spirit of laws, policies, etc. And in most of the cases for policies, there are no details of how to implement.”- A multi-lateral agency representative in KII*

### Needs and challenges of PWID/PWUD

The age range of the PWID/PWUD participants in this study was 23 to 47 years old. The participants expressed similar needs and concerns, although PWID faced higher risks of health hazards and physical abuses compared with PWUD. PWID were beaten on the street by their peers, gangsters, and police officers. Thus, they had to hide and run all the time. They reported that they were quite vulnerable to physical abuses, especially when they stole for food and drugs or when they were detained at rehabilitation centers. On the contrary, some PWUD reported that their community members and Sangkat/commune officials did not discriminate or abuse them much. This was because many villagers in their community used drugs, and people understood each other and oftentimes authorities tolerated them.

One of the main needs of PWID/PWUD was security and safety. Of the PWID participants, about 80% reported that they had no shelter and slept on the street. In fear of getting abused by gangs or the authorities, they said they carefully found a place to sleep on the street, where they could not be seen by the public or police officers.

Similar to PWID, PWUD reported that they did not feel safe to travel in the evening or late evening. All of them expressed their frustration toward and fear of police officers that usually mistreated them on the roads during their night patrols.

*“We were often abused at night by some gangsters or police officers if we were found sleeping close to their area… [We need to find a far-away place to sleep].”- PWID in FGD*

*“80% of police officers were bad and always mistreated us when they met us on the roads…They took our valuable property or beat us before they let us go.”- PWUD in FGD*

In addition to mistreatment by police officers, a high level of discrimination against PWID/PWUD was reported in the study. The majority of participants admitted without reservation that PWID/PWUD were discriminated against in their living, working, traveling, or even sleeping conditions.

*“People do not want to meet PWID/PWUD, nor want to live next to them…When I saw them on the street, I had to avoid passing them.”- A Sangkat/commune official in FGD*

During the discussions, PWUD showed more enthusiasm about the prospect of their future in relation to jobs or family in comparison to PWID. Most PWID in our study reported living alone on the street. They admitted being jobless and stealing at times. Only one of them earned a living by working at car parking areas. Many PWID used to receive vocational training from NGOs. However, they could not find a job due to their drug-injecting status. Conversely, most PWUD in our study lived in a house or shelter with their families. Physically, they looked healthier than PWID. Most of them earned a living by picking up morning glory from a lake near their community and selling it at a market. The lack of comprehensive services made it challenging to respond to the wide needs of this population. For instance, there was no clear follow-up support after being released from the rehabilitation centers. Most PWID/PWUD were released into the custody of their family without the provision of adequate information or support on further treatment needs or employment.

Many of the PWUD participants reported that they had never known about any legal aid services that reached out into their community. However, there were examples of case-by-case legal assistance provided by some non-governmental agencies. For instance, when there was abuse, calling to the Cambodian Network of People who Use Drugs (CNPUD) could help in seeking legal aid.

*“We want any NGO to lead us in protecting our rights because police will not arrest NGOs. Also, we want them (NGOs) to assist us in legal support when we are arrested by police.”- PWUD in FGD*

Some services supported and coordinated by either the government agencies (i.e., NACD and other health, education, and social agencies) or NGOs were available, but at times barely accessible or affordable. For instance, there is only one MMT clinic in the country that was run by the MoH, making it financially challenging for PWID to receive MMT services regularly. Many PWID said that NSP did not provide enough syringes because some of their peers borrowed syringes from each other. This made them extremely vulnerable to health hazards, such as HIV infection.

Rehabilitation centers have not provided a good response to the needs of PWID/PWUD, lacking a comprehensive package of services and often resulting in physical abuses.

*“…It is better to be imprisoned for a year at Prey Sar (prison) than staying in the rehabilitation center [he’s referring to a government-run center in Phnom Penh] for 3 months. I got beaten almost every day by other PWID/PWUD in the center.”- Some PWID in FGD*

Authority participants reported that most centers also charged PWID/PWUD or their family for the services provided in the centers, and some PWID/PWUD could not afford to pay for a “good” (private) center where more comprehensive services were available.

*“A PWUD aged about 30 years old was sent to a center. After six months, he returned home. He looked healthier. However, after a while, he relapsed and started using drugs again after meeting his friends. His mother is very poor, and now keeps asking the village chief to look for a center, which is free of charge.”- Some Sangkat/commune officials in FGD*

### Factors enabling mistreatment of PWID/PWUD

There are different levels of policing from the national to the local levels. At the national level, besides the NACD, an anti-drug department under the MoI has a nationwide scope of work over the Provincial/Municipal Anti-Drug Office that operates under their respective province/municipality. Also, the Khan/district and Sangkat/commune police directly handle drug issues in their respective territory. For Cambodia’s judicial procedures, in the court, first instance, the police have the primary role to detain the suspects and compile evidence before submitting a case to the court for a full investigation and judgment hearing. The many layers of policing, from the commune up to the provincial, if not national, levels, create overlapping roles among police authorities and multiple opportunities for detention of PWID/PWUD.

PWID/PWUD in the FGDs reported that police officers were active in arresting and detaining them in police custody for hours or a couple of days without any reason. However, in most cases, possession of a couple of tablets of drugs and carrying syringes were used as strong evidence for arrest because the definition of drug trafficking and drug use in the DCL and VCSP is vague. Thus, police often use possession of drugs and syringes as indications of arrest for drug use. About 70 and 80% of PWID and PWUD, respectively, reported that they were arrested in such scenario.

*“About 70% of police officers used to arrest us when we just carried a single syringe, and sometimes used violence on us during the detention…”- PWID in FGD*

Others who participated in the interviews and FGDs, including NGO staff working on harm reduction, believed that there were some cases of PWID/PWUD being arrested by the police without critical evidence of a crime or violence. Also, the vague definition of drug trafficking made law enforcement officials implement the DCL and VCSP based on a context and often criminalize drug use. According to NGO participants, the government argued that they followed an example of such a suppressing practice from neighboring countries.

*“Before, because of its unclear definition, the translation and enforcement of the law was based on each context, which was not consistent and often put drug use in a criminal definition. From the government side, they always argued that the evidence of the adoption of such a law was from other neighboring countries, such as Thailand and Vietnam.”- An NGO representative in KII*

Another greatest challenge of law enforcement was the inadequate knowledge about services to which the police could refer detained PWID/PWUD. According to the police and most government officials in the study, police had an intention to refer PWID/PWUD to detoxification services, healthcare or psychological/mental health treatment, or a rehabilitation center. But in most cases, they ended up detaining them due to the absence of knowledge about related services or programs.

*“There was a huge lack of supporting services, such as referral, treatment, and education/training. I would personally recommend representatives of the ministries under the NACD… work together to have a comprehensive service package for drug users.”- A government representative in KII*

Participants shared their observations on the impacts of the VCSP, saying that soon after the police started implementing the VCSP, most PWID/PWUD tried to hide themselves or moved to another location as they feared an arrest. PWID/PWUD perceived a stronger likelihood of arrest compared to the previously normal practices. This was an essential obstacle for harm reduction programs, particularly outreach activities.

*“The main obstacle was soon after the policy (VCSP) was put in practice, most PWID and PWUD hide themselves as they felt that they were illegal and criminal according to the interpretation of the policy itself. And that was why during that time we saw only a few PWID/PWUD.”- An NGO representative in KII*

The right to a decent living has been impacted by the enforcement of the more security-focused VCSP since 2010. Police officers reported that they usually followed an order to clear the streets off any insecurity, including gambling and drug use. With limited knowledge of harm reduction, including DCL articles supporting harm reduction interventions, officers frequently mistreated PWID/PWUD. This may depict their limited understanding of the law and policy as reported by one government official in the study.

*“…People are confused about the policy and the law. There is a law (DCL) to handle drug-related issues. Policies only state a wish or will of the government to clear up drug use or gambling, but laws should be used in treating the issues…”- A government representative in KII*

## Discussion

This study presents a number of significant findings that lend support to an argument that some articles of the DCL and VCSP about treatment of PWID/PWUD are vague, and consequently, law enforcement officials did not well understand them, which led to misunderstanding and wrong implementation. Moreover, even when some harm reduction related articles are explicitly stated, law enforcement officials misunderstood, or did not follow them. Further, as a result of not knowing the legal instruments and associated rights, PWID/PWUD viewed themselves as “illegal citizens” or criminals. This perception discouraged them from making demands for rights to legal treatment and access to proper harm reduction services. There were a number of factors that contributed to the ambiguities of the DCL and VCSP and the misunderstanding of these instruments and the mistreatment of PWID/PWUD by law enforcement officials.

### Factors contributing to ambiguities of DCL and VCSP

First, the formulation of the DCL and VCSP lacked sufficient consultation with civil society organizations and beneficiaries concerned. This may have caused inadequate or incorrect stipulation about treatment of PWID/PWUD. Law enforcement officials and the general public have a limited understanding of these legal instruments in relation to drug use. Subsequently, harm reduction efforts in Cambodia have resulted in limited success due to the improper understanding and implementation of these drug-related law and policy. Research shows that at-risk communities and community-based organizations (CBOs) perform critical roles in executing successful harm reduction interventions [5]. For instance, the government in Vietnam works with CBOs on harm reduction responses since they increasingly acknowledge that CBOs better understand and have greater access to PWID/PWUD [27]. Thus, the shortcoming in dialogs with target communities and CBOs may have rendered loopholes in understanding and addressing the needs of PWID/PWUD.

Second, the gap in understanding drug use and harm reduction by law enforcement officials stems from a very slow evolution of implementation—from policy to practice—of harm reduction in Cambodia. Harm reduction programs in the Kingdom had a long and difficult evolution [11]. Before a tardy acceptance of harm reduction by the government in 2003, all key players, including DPs and NGOs, had worked very hard to advocate for the need to implement harm reduction and to adopt policies and strategies that guide the responses of harm reduction [11]. Harm reduction activities, particularly NSP interventions, had been perceived by authorities as a motivating factor for PWID/PWUD to continue using drugs rather than an intervention to reduce harms, including risks of HIV and hepatitis C infections. Despite the existing mechanisms, such as the Police-Community Partnership Initiative (PCPI) and Provincial Drug Control Committees (PDCC), the national NSP policy remained in draft form until 2012 [11]. Law enforcement officers perceive harm reduction mainly as NSP, reflected in their lack of referring PWID/PWUD to MMT and social and health services [11, 15]. Therefore, there is an ongoing need to strengthen the adoption and enabling environment of harm reduction policies in Cambodia.

Third, the misunderstanding and malpractice of the DCL and VCSP in relation to harm reduction pertained to the lack of awareness and training of law enforcement officials about these instruments and harm reduction responses. This reinforces the need for enhancing capacity and behavioral change of law enforcement officials pinpointed by earlier research [11]. The paucity in knowledge about the legal instruments, coupled with stigmatization against PWID/PWUD, leads to abuses of PWID/PWUD by these agents. This is common in many Asian countries [5]. In Cambodia, the police perceive their role as the keeper of public order, security, and safety in communities, and they distance themselves from being a contributor to harm reduction or HIV prevention [11]. Their misconception about the correlation between crime and drug consumption stems their harsh treatment of PWID/PWUD [11].

A review on harm reduction responses in Asia and the Pacific concedes that a priority should be placed on convincing authorities that it is possible to contain, and is worth controlling, HIV epidemics by properly addressing drug use [5, 18]. It is vital to change an official standpoint that HIV among PWID cannot be prevented and reduced, and that the virus will be confined to this population, which the society despises [5]. Many of the police officers in our study did not believe in the benefits of harm reduction because they did not buy the idea that PWID are at a high risk of contracting HIV, and there has been no research suggesting that harm reduction techniques actually lower the risk of HIV transmission in Cambodia. In fact, there is global consensus that “combination prevention” harm reduction methods decrease HIV prevalence among key populations. However, the link is not a natural one, but one that is shaped by the availability and performance of harm reduction measures themselves. To exemplify, a Dutch study revealed that “full participation in harm reduction programs was associated with lower incidence of HIV infection” in PWID [28]. Participation in a NSP alone did not result in lower HIV infection, but instead multiple harm reduction strategies must be incorporated to lower HIV infection [28]. The criticality of diverse and integrated elements in harm reduction programs should be stressed because many participants in our study misconceived that harm reduction and NSP were the same, rather than NSP being just one component of harm reduction.

Capacity building for law enforcement officials should go beyond improving the comprehension and implementation of the DCL and VCSP and should embody harm reduction methods. There have been many efforts to train police officers in harm reduction. For instance, a National Harm Reduction Curriculum for the Cambodian Police Training Academy has been implemented since 2011 [29]. This initiative has yielded some positive results, such as increase in knowledge about law enforcement approaches and support of harm reduction programs. Moreover, some NGOs have provided short training courses on harm reduction to police officers, which have changed their attitudes toward harm reduction approaches and rendered a better environment for service delivery [11]. However, police officers in our study depicted a limited understanding about harm reduction approaches. Thus, more attention should be devoted to training methods and follow-up on enforcement. A study in the USA disclosed that training police officers in HIV transmission, harm reduction strategies (including NSP), and occupational safety (including the risk of contracting HIV from needle-stick injuries) enhanced their proper understanding and practices of drug-related laws [30]. Prior to the training, 51% of police officers believed NSP promoted drug use, 38% thought NSP failed to reduce HIV spread, and only 7% accurately understood the syringe possession law [30]. In the wake of the training, they were more likely to state proper interpretations of laws pertaining to PWID and understand information about occupational safety related to HIV transmission [30]. This suggests that police officers may be more inclined to learn from training that offers information about their personal health and safety and replicate it in law enforcement with PWID. The study concluded that “using occupational safety content as a vehicle to deliver a broader set of public health content” can ensure that implementing officers are knowledgeable about harm reduction methods and benefits and apply them in law enforcement [30].

Fourth, the emphasis on the physical dimension of harm reduction by seconding its social and health tenets by the DCL and VCSP and the relevant authorities exacerbated the wrong understanding and implementation by enforcement officers. This inadequate focus on social and health aspects of harm reduction implies a lack of “combination prevention” embrace of safety and security. The VCSP defines “safe communities” as those with no thefts, gambling, drug use, prostitution, or other crimes [9]. This policy presses local law enforcement officers to regard drug use as an issue of social security or safety indicated by an absence of physical crimes [11]. Not only authorities but also local communities prioritize physical security of their neighborhood over HIV prevention, which is deemed as more a matter of individuals [11]. Nonetheless, harm reduction encompasses mitigation of both physical and mental harms by providing health and social services to at-risk individuals, notably PWID/PWUD. Prioritizing the state of security over harm reduction, which is strongly associated with HIV prevention in general, may yield a short-term physical order and peace, but a long-term socio-economic repercussion in communities.

As aforementioned, to ensure “safe communities,” people could report such illicit activity as drug use to local authorities or parents could turn in their drug-using children to the police for rehabilitation. This sort of “community policing” could create fear and suspicion among villagers, which could exacerbate the post-trauma lack of mutual trust culminating from the genocidal regime (1975–1979) in the recent history of Cambodia [31].

Finally, the lack of harm reduction facilities and services and insufficient knowledge about these facilities and services made it difficult for law enforcement officials to refer PWID/PWUD for help and support. Further, the available facilities were difficult for PWID/PWUD to access, and experiences of being treated badly in the rehabilitation facilities scared them away. Also, comprehensive rehabilitation services, usually private ones, are not affordable for PWID/PWUD or their families. Services at the government rehabilitation centers were not user-friendly, and the centers were filled with physical abuses [22, 23]. A Vietnamese study has found that negative attitudes toward drug use and compulsory detention of PWID/PWUD failed to reduce HIV infections among PWID. Harm reduction strategies were not effective because the country viewed drug use as a social danger and jailed PWID/PWUD in rehabilitation centers. Many Vietnamese felt that NSP, particularly methadone treatment, supported drug use. Mandatory detention centers were plagued with abuses and unsuccessful rehabilitation. PWID/PWUD undertook many risky behaviors to avoid law enforcement out of fear of detainment [6]. Vietnam later shifted to a local harm reduction approach by changing the negative public opinion about drug use within communities and became successful in lessening HIV infections among PWID [6]. This type of strategy is likely to be successful in Cambodia as well because changing the government officials’ mindset is more challenging than focusing on community opinion.

Malaysia represents a success story in terms friendly service delivery for PWID/PWUD by moving away from a punitive approach to a voluntary, rights-based approach to drug treatment [32]. The country has transformed eight compulsory detention centers into voluntary cure and care centers or clinics [33]. Opioid substitution therapy provision in prisons increased from one site in 2008 to 18 in 2013 [34], and NSP site provision increased by 431 between 2012 and 2014 [32, 35]. It was expected that this increasing implementation of services would reduce the prevalence of PWID living with HIV [34]. It is of note that in both Vietnam and Malaysia, heroin and opiates are more prevalent, whereas in Cambodia, they are not, and this makes drug treatment and harm reduction more challenging because of a lack of evidence for approaches to helping people with dependence on ATS drugs. Contingency management and financial incentives (such as conditional cash management) are possibly the only approach for which there is evidence of success [36, 37]. Thus, Cambodia needs to consider adopting this evidence-based approach to harm reduction.

In Cambodia, it was estimated that in 2010 only 1% of PWID/PWUD in drug detention centers were admitted on a voluntary basis [22]. Methadone was not available at these centers since the majority of those admitted used ATS. These centers were essentially just prisons for detaining PWID/PWUD or people with psychiatric problems [22]. Having more harm reduction strategies implemented in prisons will mitigate the risks of the target population as many PWID are jailed for drug and non-drug related crimes.

Improving these aspects would better address the major needs of PWID/PWUD concerning safety, security, stigmatization, discrimination, and access to and affordability of harm reduction services. PWUD were more hopeful than PWID about their future regarding jobs or family. Although PWID/PWUD in our study expressed similar needs and concerns, their risks occur based on the situation or context they are in. Therefore, they might face different challenges depending on their risks [38]. “Social context, comprising interactions between individuals and environments” induces different types and levels of harm and risks [29]. Put another way, “social situations and environments” perform an integral role in determining what and how PWID/PWUD encounter risks [38].

### How punitive policies hamper harm reduction responses

The mistreatment of PWID/PWUD by arresting and detaining them mirrors a punitive practice of tackling drug use, which contradicts a rights-based approach effective in dealing with drug-related issues worldwide. Evidence depicts that punitive policies and practices exacerbate drug use and consequent ills [2–4]. For instance, a study in India revealed that negative social perceptions about and harsh treatment of PWID/PWUD increased the risk of HIV transmission [14]. This occurred since PWID/PWUD engaged in more risky behaviors, such as not carrying clean syringes/needles from NSP out of fear of getting caught with them and then punished by law enforcement officers [14]. This is also true in Cambodia because our study and an earlier study in Cambodia [15] unveil that PWID/PWUD were fearful of sanctions from law enforcement, thus not accessing harm reduction and health services.

Moreover, if PWID/PWUD in Cambodia continue to be ill-treated by law enforcement officers and rehabilitation facilities, there will be more adverse ramifications, such as more drug use and subsequent health and social problems, for the society as a whole. Cracking-down policies and measures are not effective to ensure drug control and the safety of communities. Mistreated PWID/PWUD are unlikely to openly seek safe and sterile equipment for drug injection and other support, which will amplify HIV spates. Conversely, voluntary, right-respected services will contain drug use and its consequent effects, particularly risks of contracting and spreading HIV.

### Limitations of the study

There were some limitations in this study. The first limitation concerned the limited geographic coverage that confined to three Khans/districts of Phnom Penh. Secondly, the PWID/PWUD participants were limited to those with connections to the NGO participants, meaning those without these linkages were not able to participate. PWID/PWUD in Cambodia are “sensitive” people since they are perceived as “criminals” [39]. Thus, it was difficult to recruit PWID/PWUD into the study. Sensing the difficulty, we went straight to our partner NGOs working on harm reduction to recruit the participants. The fact that they were beneficiaries of the NGOs enabled them to feel confident in partaking in the study although their views might be different from those of non-beneficiaries of the NGOs. Thirdly, given 14 of the 18 PWID/PWUD participants were males, the findings of their perceptions and experiences might have been shaped by their gender. These findings thus should be interpreted in the context of gender imbalance since drug use among women is highly prevalent.

Fourthly, the NGO staff were field staff working directly with PWID/PWUD; although, they were assigned to the study by their superiors. They were chosen because they had more knowledge on the ground than program managers; we did not know if they were chosen in a biased way by their superiors. Fifthly, we could not conduct the planned FGDs with PWID/PWUD in rehabilitation centers due to the restriction and assumption by the center management that they were not conscious enough to provide accurate responses while taking drug treatment. Moreover, it would have posed ethical challenges. Thus, their views were not presented in this study. Finally, the data collection tools (the FGD and KII guides) were not pre-tested or validated in the Cambodian context. Thus, the findings of this study should be read in lieu of these shortcomings.

## Conclusions

This research evinces a large gap in the understanding and application of the DCL and VCSP among the study participants. The chief factors that contributed to this gap comprised ambiguities of some articles in these instruments (the DCL and VCSP), the lack of participation by civil society organizations and beneficiaries in the development of these instruments and the slow embrace of harm reduction responses by the government. Also, the lack of awareness and training of law enforcement officials about these instruments and harm reduction responses, the emphasis on the physical dimension of harm reduction by seconding its social and health tenets by these instruments and the relevant authorities, and the lack of and difficult access to comprehensive harm reduction services caused this gap. Moreover, the shortage of knowledge about the DCL and VCSP, coupled with the high levels of stigmatization, social discrimination, and ill treatment, were key barriers preventing PWID/PWUD from claiming their rights, accessing needed health and non-health services, and participating in relevant programs.

To refine the impacts of harm reduction programs, first and foremost, more campaigns and awareness raising are needed to change the mindset of government agencies and the public at large from treating drug use as a security issue to regarding it as a health and social one. Second, law enforcement officials should be trained more rigorously in the DCL and VCSP. Articles particularly in relation to setting out the criminalization against and penalties imposed on PWID/PWUD need to be changed so that they can be explicitly understood and properly executed. Allied with this training, sessions on HIV transmission and harm reduction methods should be incorporated. Better comprehension of the concerned legal instruments and related tools will culminate in effectual implementation in a voluntary, right-based fashion. A comprehensive package essential to respond to the needs of PWID/PWUD should encompass legal aid, treatment, and follow-up care (including family and community reintegration), livelihood support, and vocational opportunities.

Ultimately, more synergistic collaboration needs to be built between human rights or legal NGOs and harm reduction implementers to provide more tailored and effective services to PWID/PWUD. Particular attention should be paid to (1) consistent legal aid to the victims, particularly at the scene of an arrest or when detention occurs, (2) cooperation with law enforcement officials to protect the rights of PWID/PWUD who are being arrested, (3) necessary socio-economic assistance to PWID/PWUD, and (4) increasing the quality of services at both private and government rehabilitation centers.

## Abbreviations

AAHR: Asia Action on Harm Reduction
AIDS: Acquired Immunodeficiency Syndrome
ATS: Amphetamine-type stimulants
CCHR: Cambodian Center for Human Rights
CNPUD: Cambodian Network of People Who Use Drugs
DCL: Drug Control Law
DFAT: Department of Foreign Affairs and Trade
DMHSA: Department of Mental Health and Substance Abuse
EC: European Commission
FEW: Female entertainment worker
FGD: Focus group discussions
GFATM: Global Fund to Fight AIDS, Tuberculosis and Malaria
HAARP: HIV/AIDS Asia Regional Programme
HIV: Human immunodeficiency virus
KII: Key informant interview
KP: Key populations
MMT: Methadone Maintenance Therapy
MoH: Ministry of Health
MoI: Ministry of Interior
MSM: Men who have sex with men
NAA: National AIDS Authority
NACD: National Authority for Combating Drugs
NCHADS: National Center for HIV/AIDS, Dermatology and STD
NECHR: National Ethics Committee for Health Research
NGO: Non-government organization
NSP: Needle and syringe program
PCPI: Police-community partnership initiative
PDCC: Provincial Drug Control Committees
PWID: People who inject drugs
PWUD: People who use drugs
RGC: Royal Government of Cambodia
TG: Transgender
UN: United Nations
UNAIDS: United Nations Joint HIV/AIDS Programmes
UNODC: United Nations Office on Drug and Crimes
VCSP: Village-Commune Safety Policy
WHO: World Health Organization
